# Bipedicle Frontal-Occipital Flap for Reconstruction of Post Avulsion Injury of Scalp Temporoparietal Region: A Case Report

**DOI:** 10.29252/wjps.10.3.121

**Published:** 2021-09

**Authors:** Deepak Krishna, Manal M Khan, Michael Laitonjam

**Affiliations:** Department of Burns and Plastic Surgery, All India Institute of Medical Sciences, Bhopal, India

**Keywords:** Temporo-parietal region, Avulsion injury, Bipedicle fronto-occipital flap

## Abstract

The reconstruction of the scalp following avulsion injury has always been a great challenge for plastic surgeons. Here we report a 25 yr old female presented with necrosis of left temporoparietal scalp skin over left temporo-parietal region following history of avulsion injury of the scalp four days back at all India Institute of Medical Sciences, Bhopal, India in 2018. After removal of the necrosed skin, the defect was successfully covered with Bipedicle fronto-occipital flap.

## INTRODUCTION

The reconstruction of the scalp following avulsion injury has always been a great challenge for plastic surgeons. Although rare, scalp avulsion injuries mostly occur in working places and in female patients, it is often caused because of accidents related to their comparatively longer hair^1^.

Selection of the flap for reconstruction either local or free depends on the location, size of the defect, and timing of surgery. Small and medium-sized scalp defects easily covered by a single local unipedicle flap but large and complex scalp defects usually required multiple local flaps, microvascular free tissue transfer for coverage, or microsurgical replantation. 

No doubt unipedicle flaps are vascularised, versatile flaps used for reconstruction of various scalp defects, however, the distal portion of unipedicle pericranial flap has no definite axial blood supply, and no connection with the contralateral vessels^2^. 

Here, we report a case of post avulsion injury of the temporoparietal region of the scalp which was reconstructed by using the bipedicle frontal-occipital flap.

## CLINICAL DETAILS

**Fig.1:**
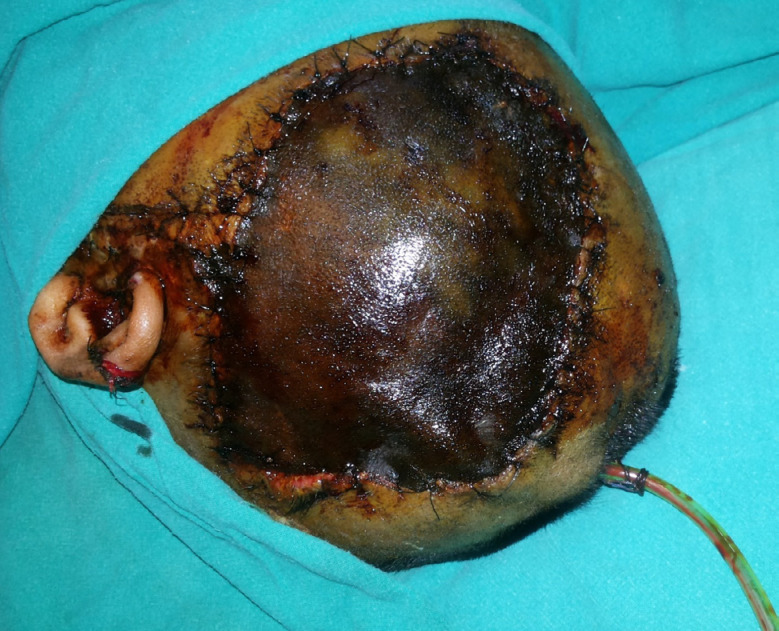
Pre-operative image showing blackening sutured scalp

A 25 yr old female presented with necrosis of left temporoparietal scalp skin (Figure 1) at All India Institute of Medical Sciences, Bhopal, India in 2018. 

The study was approved by the Institutional Review Board and informed consent was obtained from the participant to show her clinical photographs. 

She had a history of avulsion injury of the scalp left temporoparietal region while working with a chaff cutter machine four days back. The patient initially managed at an outside hospital, where she was operated on for removal of depressed fracture segment left temporoparietal region and repair of Dural rent with superficial temporoparietal fascia of the avulsed scalp. Then, the avulsed scalp was sutured back to the defect site for temporary coverage. The sutured avulsed scalp gradually became necrosed. On examination, the dimension of necrosed skin was 18 cm x 10 cm. The patient was conscious and oriented and her general condition was stable at presentation.

## OPERATIVE DETAILS

**Fig. 2:**
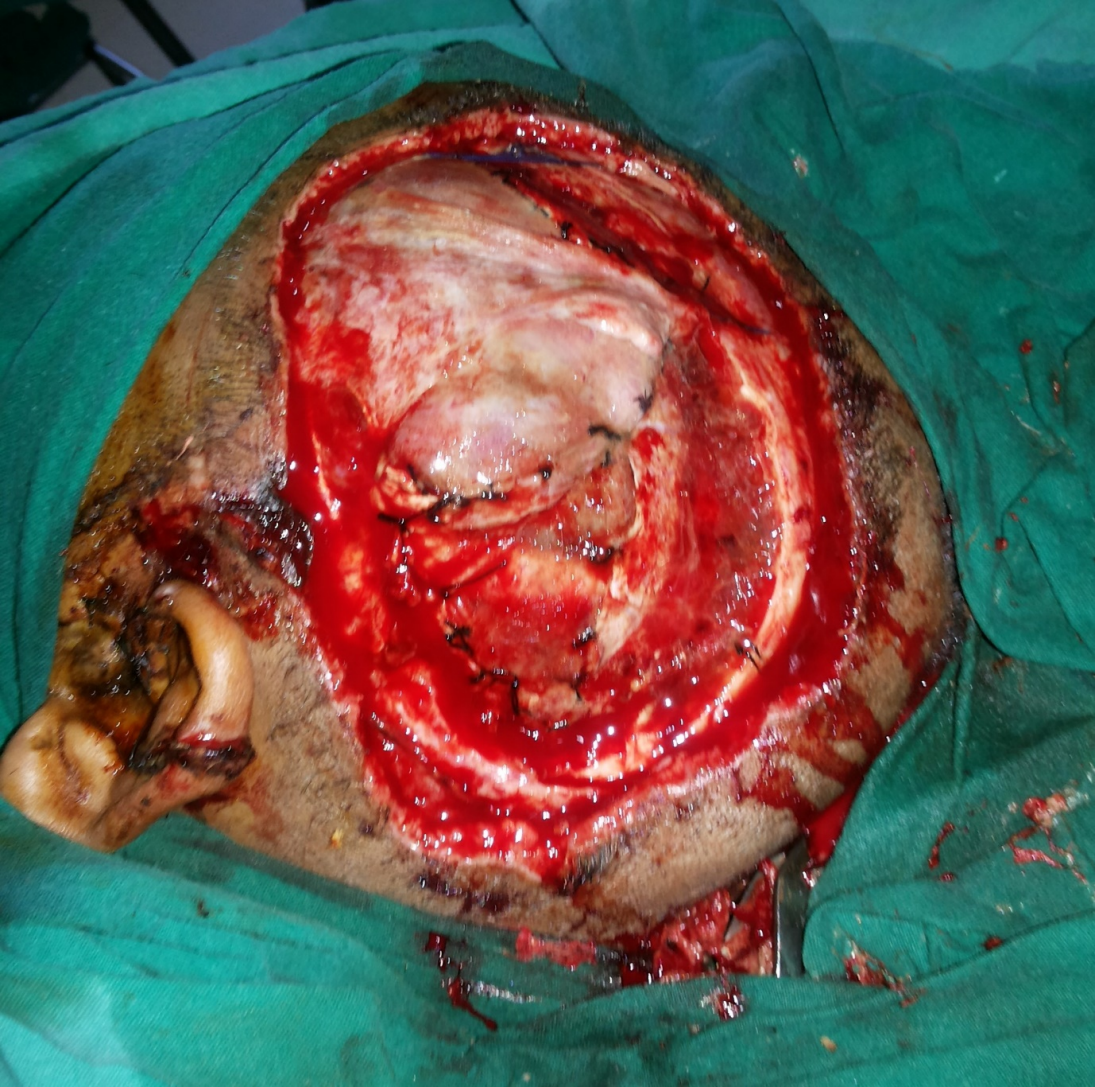
Intraoperative image showing exposed dura with loss of bone

**Fig. 3:**
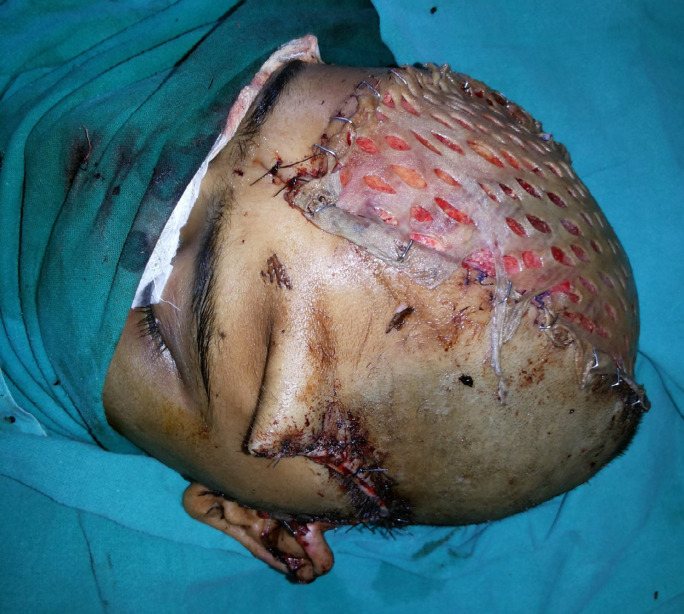
Intra-operative image showing defect covered with bipedicle fronto-occipital flap and skin graft at donor site

**Fig. 4:**
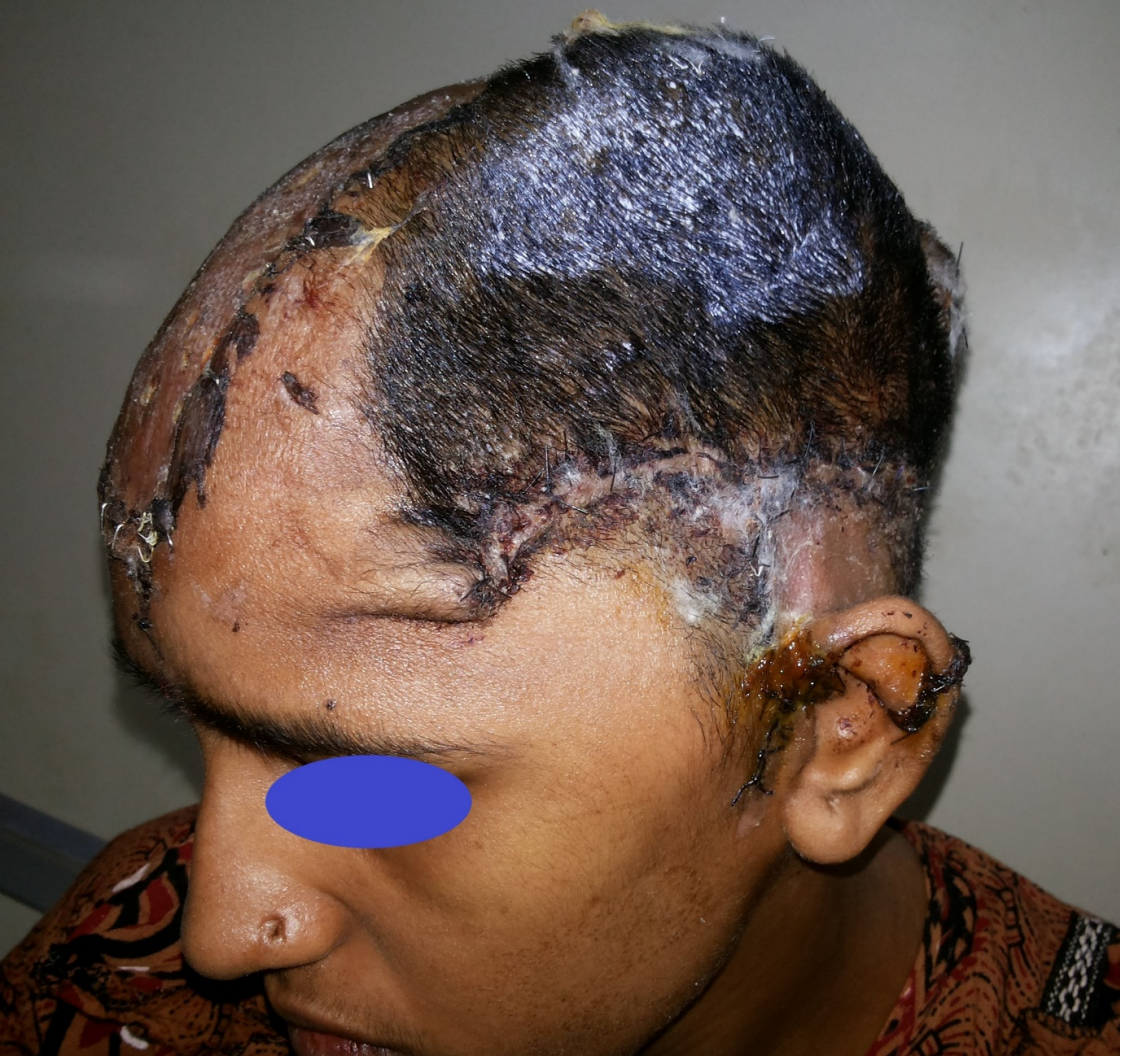
Weeks follow-up image showing well settled flap

The second surgery was planned for the removal of necrosed skin and coverage with an appropriate flap. After removal of the necrosed scalp (Figure 2) and on the exploration of the left temporal region, superficial temporal artery and veins were not found because of the trauma zone. Therefore, Bipedicle frontal-occipital flap was planned. A 26 cm x 12 cm sized bipedicle frontal-occipital flap designed to cover the defect. The flap was elevated in the subgaleal plane and transferred directly to the exposed dural layer and donor site covered with a split-thickness skin graft (Figure 3). Anteriorly the flap was based on the left supratrochlear, left supraorbital arteries, and posteriorly on the left and right occipital arteries. The flap was healthy in the postoperative period (Figure 4 at 2^nd^ week). 

## DISCUSSION

After Miller et al, reported the first successful scalp replantation^3^, microsurgical replantation has been considered the first choice in the treatment of scalp avulsion. However, this approach depends on the time of presentation of the patient and the severity of the injury.

The scalp has got rich vascular supply. It is supplied by five pairs of arteries. From anterior to posterior, they are supratrochlear, supraorbital, superficial temporal, posterior auricular, and occipital arteries.

Depending on the size of the defects, small and moderate size scalp defects can be managed with various available local flaps. But sometimes the availability of local flap is questionable and has got certain limitations in case of large defect. For a large defect, even a free flap can also be used but it might be disadvantageous, as it takes a long operating time and the relatively high donor site morbidity^4^^-^^6^.

Considering the bipedicle flap, it has got certain merits regarding its versatility, less functional complications, and donor site morbidity. Moreover, it is based on the axial pattern of blood supply from the main branches of the scalp and their perforators^7^.

There have been few cases where Bipedicle flap has been reported for use in post oncological defects^8^, post-electric burn scalp defect^2^ post-osteomyelitis scalp defect^9^, etc.

## CONCLUSION

Considering from the above case, bipedicle flap whether frontal-occipital or temporo-temporal can be used for extensive soft-tissue defect of the scalp.

## CONFLICT OF INTEREST

The authors do not have any conflicts of interest to disclose in relation to this case.
